# Growth and Differentiation of the Larval Mosquito Midgut

**DOI:** 10.1673/031.009.5501

**Published:** 2009-07-15

**Authors:** Kathryn Ray, Maria Mercedes, Doris Chan, Chi Yan Choi, James T. Nishiura

**Affiliations:** Biology Department, Brooklyn College, 2900 Bedford Ave, Brooklyn, NY 11210

**Keywords:** metamorphosis, Aedes aegypti, Culex pipiens, BrdU

## Abstract

Factors affecting larval growth and nutrition have consequences on adult fecundity. Since the mosquito larval midgut is the primary organ of digestion and nutrient absorption, factors that affect the growth and development of the midgut may have potential consequences on the reproductive potential of the adult. To gain a better understanding of mosquito midgut development the growth and metamorphic remodeling of the *Aedes aegypti* L. and *Culex pipiens* L. (Diptera: Culicidae) midguts were investigated. Cytological evidence was obtained suggesting that, in both the anterior and posterior *Ae. aegypti* larval midgut, diploid regenerative cells give rise to new endoreplicating cells that significantly contribute to the growth and metabolism of the midgut. This hypothesis was supported by BrdU incorporation studies showing that diploid cells, as well as large and small endoreplicating cells, synthesize DNA during the 2^nd^, 3^rd^ and 4^th^ instars. Cytological studies of the *Cx. pipiens* larval midgut suggest that anterior midgut growth in this species is primarily by cell enlargement. To study metamorphic remodeling of the midgut, DNA synthesis in *Ae. aegypti* 4^th^ instar midguts was followed by using 5-bromo-2-deoxyuridine (BrdU) incorporation. During the 24 hr period after the last larval-larval molt both endoreplicating and diploid cells incorporate BrdU. After the critical weight is achieved, endoreplicating cell BrdU incorporation gradually ceases while diploid cells continue to replicate. The period of maximum diploid cell incorporation correlated with the period of maximum ecdysone titer.

## Introduction

Factors controlling the growth and nutritional state of insect larvae affect the reproductive potential of the adult (Chambers and Klowden 1994; Soliman et al. 1995; Tu and Tatar 2003; Zhou et al. 2004). Small, poorly fed mosquito larvae produce adults of reduced reproductive potential (Briegel 1990; Renshaw et al. 1994; Briegel 2003; Noriega 2004; Telang and Wells 2004; Telang et al. 2006; Telang et al. 2007). Interfering with the normal development of the midgut might reduce the larva's ability to absorb, or store, nutrients and, as a consequence, reduce adult fecundity. Thus detailed knowledge of regulators of midgut development could identify targets that may be modulated to affect female fecundity and control mosquito populations.

The midgut is composed of an epithelial layer of large endoreplicating cells that have been identified as having polytene chromosomes (Trager 1937; Sutton 1942; Gillham 1957; Clements 1992). Interspersed among the endoreplicating cells are diploid regenerative cells (Trager 1937; Berger 1938; Richins 1945). The endoreplicating cells of the midgut perform functions such as ionic and osmotic regulation (Clements 1992), lipid and carbohydrate storage (Wigglesworth 1942; Clements 1992; Nishiura et al. 2007), control of the midgut lumen pH (Corena et al. 2002; Seron et al. 2004) and secretion of digestive enzymes and absorption of nutrients. In addition, the midgut has associated endocrine (Brown et al. 1986; Brown and Cao 2001; Moffett and Moffett 2005) and muscle cells (O'Brien 1965; Bernick et al. 2007). By measuring larval length, and the length of the anterior midgut epithelial cells, Trager (1937) concluded that *Aedes aegypti* L. (Diptera: Culicidae) midgut growth was, in large part, a result of cell enlargement and not cell proliferation. However little else is known concerning the development of the mosquito larval midgut and factors that control its growth.

Metamorphosis of the mosquito midgut is a remodeling process in which larval endoreplicating cells undergo programmed cell death while imaginai diploid cells replicate giving rise to the adult midgut epithelium. During the 4 (final) larval instar, midgut diploid cells increase in number, in both the anterior and posterior midgut regions. Most of the increase in diploid cell number occurs in the posterior midgut. Shortly before pupation, a decrease in larval size and weight is accompanied by a shortening of the midgut and endoreplicating larval cells start to detach from the basement membrane and slough off into the midgut lumen. The discarded endoreplicating cells eventually form the yellowish meconium of the pupal midgut (Richins 1938; Nishiura and Smouse 2000; Nishiura 2002).

The hormonal and transcriptional processes that control mosquito metamorphosis and midgut remodeling are beginning to be understood (Jenkins et al. 1992; Nishiura et al. 2003; Nishiura et al. 2005; Margam et al. 2006; Parthasarathy and Palli 2007). The critical weight for metamorphosis occurs by 24 hours after the last larval-larval molt, suggesting that by this time JH concentrations are low enough so that a subsequent increase in ecydysone concentration will initiate metamorphosis (Chambers and Klowden 1990; Nishiura et al. 2007; Telang et al. 2007). There is a biphasisc increase in ecdysone concentration after the critical weight is achieved that very likely initiates metamorphosis (Jenkins et al. 1992; Lan and Grier 2004; Margam et al. 2006; Telang et al. 2007).

Both diploid cell division and endoreplicating cell removal appear to be affected by JH concentrations since the application of juvenile hormone analogues to 4^th^ instars affects both processes. High concentrations of JHA interfere with both diploid cell division and removal of endoreplicating cells. At lower concentrations, diploid cell division occurs but endoreplicating larval cells are not completely removed from the basement membrane (Nishiura et al. 2003; Nishiura et al. 2005; Wu et al. 2006; Parthasarathy and Palli 2007).

In this communication are reported cytological investigations into the growth and metamorphosis of the mosquito larval midgut of two species, *Ae. aegypti* and *Culex pipiens.* Addressed are two questions related to the process of cell division in the larval midgut. Does growth of the larval midgut occur only by cell enlargement? Secondly, when during midgut metamorphosis do diploid cells, that make-up most of the adult midgut, replicate? Results presented here suggest that during the period of larval growth newly formed endoreplicating cells arise in the *Ae. aegypti* anterior and posterior midgut. This process appears to be somewhat different in larval *Cx. pipiens* in that few newly formed endoreplicating cells are observed in the anterior midgut, but many are observed in the posterior midgut. In both species, newly formed endoreplicating cells appear to eventually make-up a substantial proportion of the midgut epithelial cells. To explain the origins of the newly formed endoreplicating cells, it is hypothesized that during larval growth, diploid cells divide and some differentiate into newly formed endoreplicating cells, and these cells substantially contribute to the growth of the larval midgut. If correct, growth of the mosquito larval midgut should be viewed as resulting from cell enlargement and cell division. This differs from a previous hypothesis suggesting that diploid cells give rise to endoreplicating cells only to replace those lost during the larval phase and midgut growth occurs by cell enlargement (Trager 1937). BrdU incorporation studies presented here indicate that approximately 48 hours after the last larval-larval molt, diploid cell division largely ceases in the *Ae. aegypti* anterior midgut but appears to accelerate in the posterior midgut. This cell division appears to be related to metamorphic midgut remodeling in that the dividing diploid cells persist, and are present in the pupal midgut, which eventually becomes the adult midgut. Overall, results presented here suggest that cell division may be important to both mosquito larval midgut growth and metamorphosis. An implication of this suggestion is that factors that affect cell division may have consequences on larval nutrition and adult fecundity.

## Materials and Methods

### Culture of mosquito larvae

*Ae. aegypti* eggs (Orlando strain) were obtained from Louisiana Biologicals and *Cx. pipiens* eggs were obtained from Carolina Biologicals. Larvae were grown at 24 °C and fed a mixture of TetraMin Cichlid food (8g), bakers yeast (4g) in 1.0 liter water. Under these conditions *Ae. aegypti* and *Cx. pipiens* 1^st^ instars molted approximately 48 hours after egg hatching. Second instars were identified by the shed 1^st^ instar cuticle and the clear head of a newly molted larva. The duration of the 2^nd^ instar was approximately 24 hours. Likewise, 3^rd^ instars were identified by the shed 2^nd^ instar cuticle and clear head of a newly molted larva. The duration of the 3^rd^ instar was approximately 24 hours. The collection of 4^th^ instars, at various times after the last larval-larval molt, was carried out as described previously (Nishiura et al. 2005).

### Larval midgut staining

Dissected midguts were fixed for 30 minutes at room temperature in 4% paraformaldehyde dissolved in phosphate buffered saline (PBS). They were then rinsed twice in distilled water and stained with a two-fold dilution of Mayer's hematoxylin solution (Sigma, www.sigmaaldrich.com). DAPI, 4′,6-diamidino-2-phenylindole, (Sigma), at a final concentration of 0.1 µg/ml, was added to the staining solution. After staining with hematoxylin and DAPI for 10 minutes the midguts were washed twice with water and mounted for microscopy.

The stained midguts were examined by bright field and fluorescence microscopy. Larval midguts had detectable autofluorescence when excited at 495nm and observed at 525nm using a Fluorescein isothiocyanate (FITC) filter set. The combination of hematoxylin stain and autofluorescence resulted in well delineated cell boundaries so cell and nuclear areas could be measured.

### 5-bromo-2-deoxyuridine (BrdU) labeling

First, 2^nd^ and 3^rd^ instars were exposed to 100 µg/ml BrdU (Sigma) for 24 hours at 24 °C. Midguts were then dissected and fixed overnight at 4 °C in 4% paraformaldehyde dissolved in PBS. The fixed midguts were serially equilibrated in solutions of increasing methanol concentrations (50%, 75% 100%), and then serially equilibrated in solutions of increasing ethanol concentrations (50% ethanol: 50% methanol; 75% ethanol: 25% methanol; 100% ethanol) and stored at -20 °C. Fourth instars, at various times after the last larval-larval molt, were exposed to 100 µg/ml BrdU for 8 to 12 hours and then midguts were dissected, fixed and stored as described above.

### BrdU immunohistochemistry

Incorporated BrdU was detected by the DNase digestion method (Tkatchenki 2006). Midguts, fixed and stored as described above, were washed 5X in PBS containing 0.5% tween 20. They were then washed 3X in DNase buffer (50 mM Tris, pH 7.6; 10mM MgCl2). DNase (RNase-free, Ambion, www.ambion.com), at a final concentration of 0.1 unit/µl, was added and the midguts incubated at 37 °C for 2 hours. After incubation, the midguts were washed in TBS (50 mM Tris, pH 7.6; 100 mM NaCl) and heated at 70 °C for 10 minutes to inactivate the DNase. Midguts were then washed 5X in blocking buffer (TBS-5% BSA) composed of TBS plus 5% bovine serum albumin (BSA). They were then incubated with a 1:200 dilution of mouse anti-BrdU (Sigma) for 2 hrs at room temperature. After incubation, midguts were washed 5X with TBS-5% BSA and stored overnight at 4 °C in this buffer. After overnight storage midguts were brought to room temperature and a 1:200 dilution of HRP conjugated goat anti-mouse (Sigma) was added. After incubation with secondary antibody for 2 hr at room temperature, midguts were washed 5X with TBS-5% BSA and 3X with TBS. A 4-chloro-l-naphthol based color development reagent (BioRad, www.biorad.com), or ImmPACT DAB (Vector Laboritories, www.vectorlabs.com), was added and the peroxidase reaction proceeded until distinct labeling was detected. The midguts were then washed with PBS and mounted in 50% glycerol for microscopic examination.

### Measurement of cell and nuclear area

**Figure 1.**
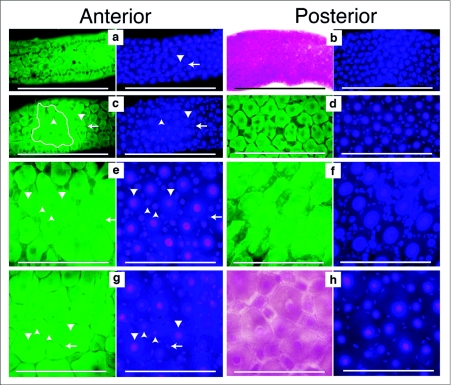
Growth and development of the Aedes *aegypti* larval midgut. Midguts from 1^st^ through 4^th^ instars were dissected, fixed and stained with Hematoxylin and DAPI. First through 3^rd^ instar midguts were dissected approximately 12 hours after the molt. Fourth instar midguts were dissected 24 hours after the molt. The left side image of each pair was taken using autofluorescence (green) or bright field (red), to emphasize the boundaries of the cells. The right side image is the same field of view showing DAPI stained nuclei. All images were taken at the same magnification and the scale bar represents 200 µm. Large nuclei are indicated by downward pointing arrow heads. Intermediate sized nuclei are indicated by upward pointing arrow heads. Small oval nuclei are indicated by left pointing arrows, (a) 1^st^ instar anterior midgut had uniformly spaced large nuclei with interspersed small nuclei, (b) 1^st^ instar posterior midgut had uniformly spaced large nuclei with interspersed small nuclei, (c) 2^nd^ instar anterior midgut had uniformly spaced large nuclei and more interspersed smaller and intermediate sized nuclei, (d) 2^nd^ instar posterior midgut had uniformly spaced large nuclei and numerous interspersed smaller and intermediate sized nuclei, (e) 3^rd^ instar anterior midgut had uniformly spaced large nuclei that were surrounded by numerous intermediate and small sized nuclei, (f) 3^rd^ instar posterior midgut had uniformly spaced large nuclei and many intermediate and small sized nuclei surrounding the large nuclei, (g) 4^th^ instar anterior midgut had large and intermediate sized nuclei surrounded by small elongate nuclei, (h) 4^th^ instar posterior midgut also had large and intermediate sized nuclei surrounded by elongate small nuclei.

To estimate the size of the anterior midgut, an area bounded by 10 adjacent cells, having the largest nuclei, was determined. An example is shown in Figure 1c. Multiple measurements per midgut were taken by measuring several 10 cell regions that overlapped by two cells. The number of small and intermediate sized nuclei, and the area encompassed by their cells, within the region bounded by the 10 large cells, was measured. These cells were designated the interstitial cells and had nuclei of various sizes, all smaller than the nuclei of the largest cells. To measure nuclear size, the areas of sharply focused, DAPI stained nuclei were determined. Cell area, as well as nuclear area, was estimated from digital images taken at the same magnification, using ImageJ software (http://rsb.info.nih.gov/ij/).

## Results

### Cytology of *Ae. aegypti* larval midgut growth

Anterior and posterior regions of 1^st^ instar midguts had uniformly distributed large nuclei of relatively equal size. There were also a few small nuclei, some of which were interspersed among the large nuclei and others arranged in long chain-like arrays (Figure 1a, b). Phalloidin staining indicated that the small nuclei arranged in long chain-like arrays may be those of muscle cells that form the contractile network along the entire midgut (data not shown). Midguts of 2^nd^ instars also had uniformly distributed large nuclei of relatively equal size in both the anterior and posterior regions (Figure 1c, d). There appeared to be more small elongate and round nuclei interspersed among the largest nuclei. Also visible were small nuclei arranged in a linear chain-like array. Third instar anterior and posterior midguts had very pronounced and numerous intermediate sized nuclei that surrounded the uniformly spaced larger nuclei (Figure 1e, f). The elongate, and smallest round nuclei, were arranged like those in 2^nd^ and 1^st^ instars. Fourth instar midguts had a mixture of large cells with large nuclei, intermediate size cells with nuclei of many different sizes, and very small nuclei surrounding the largest nuclei (Figure 1g, h).

The cytological results suggested that larval midgut growth in *Ae. aegypti* was associated with an increase in number of intermediate sized cells as well as increased cell size. The smallest nuclei, that were interspersed between the largest nuclei, were often found in pairs and mitotic figures were observed (data not shown), suggesting that the smallest cells divided during the larval stages.

### Cytology of Cx. *pipiens* larval midgut growth

**Figure 2.**
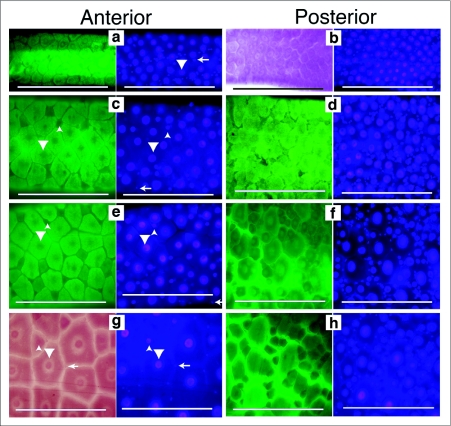
Growth and development of the *Culex pipiens* larval midgut. Midguts from 1^st^ through 4^th^ instars were dissected, fixed and stained with Hematoxylin and DAPI. First through 3^rd^ instar midguts were dissected approximately 12 hours after the molt. Fourth instar midguts were dissected 24 hours after the molt. Each subfigure has a left side image, taken using autofluorescence (green) or bright field (red), to emphasize the boundaries of the cells. The right side image is the same field of view to show DAPI stained nuclei. Large nuclei are indicated by downward pointing arrow heads. Intermediate sized nuclei are indicated by upward pointing arrow heads. Small oval nuclei are indicated by left pointing arrows. All images are taken at the same magnification and the scale bar represents 200 µm. (a) 1^st^ instar anterior midgut have evenly spaced large nuclei with a few small nuclei between them, (b) 1^st^ instar posterior midgut has evenly spaced large nuclei with more small nuclei between them, (c) 2^nd^ instar anterior midgut has evenly spaced large nuclei and a few intermediate sized nuclei between them, (d) 2^nd^ instar posterior midgut has evenly spaced large nuclei with many small and intermediate sized nuclei between them, (e) 3^rd^ instar anterior midgut has evenly spaced large nuclei with a few intermediate sized nuclei between them, (f) 3^rd^ instar posterior midgut has evenly spaced large nuclei with many intermediate and small sized nuclei between them, (g) 4^th^ instar anterior midgut has evenly spaced large nuclei, a few intermediate sized and small nuclei between them, (h) 4^th^ instar posterior midgut has many small and intermediate sized nuclei surrounding the large nuclei.

Our previous studies indicated that, unlike *Ae. aegypti,* the *Cx. pipiens* 4^th^ instar anterior midgut had few small and intermediate sized endoreplicating cells (Nishiura and Smouse 2000), suggesting that the pathway of midgut growth may differ among mosquito species. To further investigate this, midgut growth during *Cx. pipiens* larval development was cytologically investigated. *Cx. pipiens* 1^st^ instar anterior and posterior midguts had large nuclei of relatively uniform size and distribution. Smaller nuclei were either interspersed among the large nuclei or arranged in a chain-like array (Figure 2 a, b). Anterior and posterior midguts of 2^nd^ instars also had evenly distributed large nuclei. However, posterior midguts of 2^nd^ instars appeared to have many more of the smallest nuclei than did the anterior midgut (Figure 2 c, d). Third instar anterior midguts looked very different than posterior midguts. The anterior midgut had many large cells, of relatively uniform size with large nuclei, and interspersed among them were a few intermediate sized nuclei. However, posterior midguts of 3^rd^ instars had many intermediate size nuclei, heterogeneous in size, surrounding the largest nuclei (Figure 2 e, f). The anterior midgut of *Cx. pipiens* 4^th^ instars had mainly large cells with few widely separated cells of intermediate size. The posterior midgut of 4^th^ instars was similar to that of the 3^rd^ instar posterior midgut. Comparisons of larval midguts from *Cx. pipiens* and *Ae. aegypti* suggested that the posterior midguts of the two species grew in a very similar manner. Anterior midgut growth appeared very different in the two species.

### Measurement of A. *aegypti* and Cx. *pipiens* midgut growth

To estimate midgut growth only anterior midgut cells were measured since 3^rd^ instar posterior midgut cells were too numerous and closely packed to accurately determine their number. Areas of anterior midguts bounded by 10 clearly defined adjacent cells having the largest nuclei were measured (Figure 1c). It was assumed that the largest cells, with the largest nuclei, would be representative of the oldest cells of the midgut. Multiple measurements from a midgut were taken by measuring several large cell regions that overlapped by two cells. The contribution of small and intermediate sized cells to midgut growth was estimated by counting the number of intermediate and small nuclei within the areas delineated by 10 large cells. The areas encompassed by the cells having these small and intermediate sized nuclei were also measured. Nuclei were classified into four groups: (1) muscle cell nuclei that were identified by their arrangement in chain-like arrays; (2) small irregular or oval shaped nuclei, approximately equal in size to the muscle cell nuclei but interspersed between the larger nuclei; (3) intermediate sized nuclei, circular in shape but larger than the muscle cell nuclei and interspersed among the larger cells; and finally (4) the largest nuclei that were surrounded by the other nuclei.

**Figure 3.**
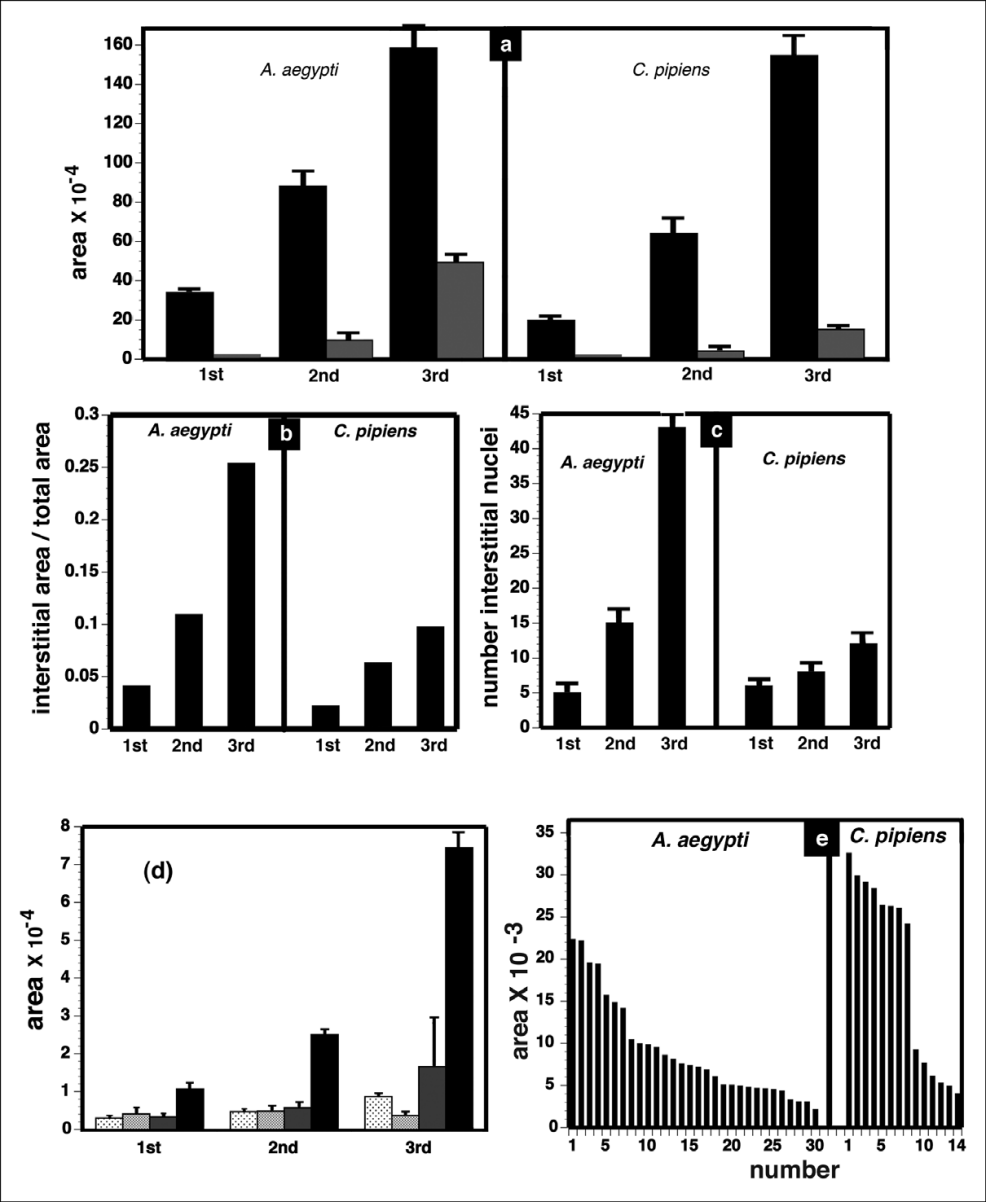
Measurements of Aedes *aegypti* and *Culex pipiens* midgut growth. Bright field and fluorescence images of midguts were used to measure growth in 1^st^ through 3^rd^ instar midguts that were dissected 12 hours after the molt. The areas of anterior midgut regions bounded by 10 adjacent cells with large nuclei were measured (black bars). An example is shown in Figure 1c. The areas of interstitial small and intermediate sized cells within the 10 cell regions were measured (grey bars). The number of interstitial small and intermediate sized nuclei within the 10 cell regions were counted. Areas are measured in pixels using ImageJ software, (a) Growth of anterior midgut from 1^st^ to 3^rd^ instars. Ten cell area (black bars); interstitial small and intermediate sized cell area (grey bars), (b) Fraction of anterior midgut growth due to interstitial small and intermediate sized cells, (c) Number of interstitial small and intermediate cell nuclei, (d) Sizes of nuclei in Ae. *aegypti* 1^st^ through 3^rd^ instars. Nuclei identified by their chain-like arrangement and circular shape (stippled bars). Small oval nuclei interspersed between larger nuclei (crosshatched bars). Intermediate sized nuclei, round in shape, and interspersed between largest nuclei (grey bars). Largest nuclei surrounded by small and intermediate sized nuclei (black bars), (e) Distribution of cell sizes in anterior midguts of Ae. *aegypti* and *Cx. pipiens* 4^th^ instars. Squares with the dimensions of 0.3mm long by 0.3mm wide were marked in autofluorescence images of Ae. *aegypti* and *Cx. pipiens* 24 hour postmolt 4^th^ instar anterior midguts and the sizes of the cells within these squares were measured.

Measurements of larval anterior midgut growth were taken in 1^st^, 2^nd^ and 3^rd^ instars. During this time period, the areas bounded by 10 adjacent large cells increased approximately 5 fold in *Ae. aegypti* (Figure 3a) and approximately 4 fold in *Cx. pipiens* (Figure 3 a). The areas encompassed by the small and intermediate sized cells, within the boundaries of 10 large cells, increased approximately 33 fold in *Ae. aegypti* (Figure 3 a) and 10 fold in *Cx. pipiens* (Figure 3a). The number of small and intermediate sized nuclei, within the boundaries of 10 large cells, increased approximately 8 fold in Ae. *aegypti* (Figure 3c) and 2 fold in *Cx. pipiens* (Figure 3c). The results indicated that, in 3^rd^ instars, the intermediate and small sized cells contributed approximately 25% of the *Ae. aegypti* anterior midgut area (Figure 3b) and approximately 10% of the *Cx. pipiens* anterior midgut area (Figure 3b).

In *Ae. aegypti,* nuclei arranged in long chains, and small oval shaped nuclei interspersed among the large nuclei, remained relatively constant in size from the 1^st^ to 3^rd^ instars (Figure 3d). The average size of the intermediate sized nuclei increased approximately 3 fold during this time period. However there was a large variation in sizes, ranging from 2 to 10 times the size of the smallest cell nuclei. The average size of the largest nuclei increased approximately 7 fold during this time period. In 3^rd^ instars there was about a 2 fold variation in size of the largest nuclei (Figure 3d). The increase in number and area of intermediate sized cells during the interval between the 1^st^ to 3^rd^ instars suggested that they differentiated and grew during this time period.

Anterior midguts of *Ae. aegypti* 4^th^ instars appeared to be a mosaic of cells of different sizes (Figure 1g) while anterior midguts of *Cx. pipiens* 4 instars appeared to be mainly composed of large cells with intermediate sized cells interspersed among them (Figure 2g). To get an estimate of the distribution of cell sizes in the anterior midguts, areas of equal size in the anterior midguts of both species were outlined and the size distribution of cells within these areas was determined (Figure 3e). There was approximately a 5 fold difference in cell sizes in *Ae. aegypti* anterior midgut and the distribution of sizes was relatively continuous. In *C. pipiens* anterior midguts there was approximately a 6 fold difference in cell sizes which appeared to be arranged into two groups: a large sized cell group and an intermediate sized group.

### BrdU incorporation in *Ae. aegypti* 2^nd^ and 3^rd^ instars

**Figure 4.**
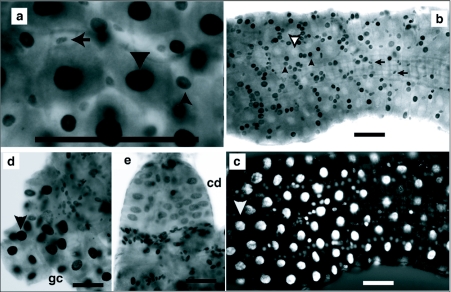
Endoreplicating and diploid cells of 2^nd^ and 3^rd^ instars incorporate BrdU. Aedes *aegypti* 1^st^ through 3^rd^ instars were exposed to 100µg/ml BrdU for 24h. BrdU labeled large nuclei are indicated by solid downward pointing arrow head. Large nuclei not labeled with BrdU are indicated by open downward pointing arrowhead. Intermediate sized nuclei are indicated by upward pointing arrow head. Small oval nuclei are indicated by left pointing arrow. Scale bar represents 100 µm. (a) Anterior midgut of 2^nd^ instar showing small oval, intermediate and large sized nuclei were BrdU labeled, (b) Posterior midgut of 3^rd^ instar showing small oval and intermediate sized nuclei were labeled by BrdU. Large nuclei were not labeled, (c) Same field of view as presented in Figure 4b illuminated to reveal DAPI stained nuclei. Large nuclei are brightly fluorescent but unlabeled by BrdU. (d) Gastric caecum (gc) of 3^rd^ instar showing labeled large nuclei and labeled diploid nuclei at the base of the gastric ceacum. (e) Cardia (cd) of 3^rd^ instar showing labeled large nuclei and small nuclei at the base of the cardia.

Cells with intermediate size nuclei may have differentiated from mitotically dividing regenerative diploid cells. To determine if small, intermediate and large cells replicated their DNA during the larval stages, BrdU incorporation experiments were performed. First, 2^nd^ and 3^rd^ instars were exposed to BrdU for 24 hours, during which time some of each instar molted. Labeling in this manner should have exposed premolt, intermolt, and newly molted larvae to BrdU and allowed the detection of DNA synthesis even if it only occurred at specific intervals during an instar. The incorporation data indicated that in 2^nd^ instars the very smallest, as well as large and intermediate size cells incorporated BrdU (Figure 4a). In some 3^rd^ instars, the largest nuclei were unlabeled, while the surrounding intermediate size nuclei and smallest nuclei were labeled (Figure 4b, c). In other midguts the largest nuclei were labeled but the surrounding intermediate size nuclei were mostly unlabeled (data not shown). These patterns suggest that DNA replication in the largest and intermediate size cells did not occur during the same time periods. Extensive DNA synthesis was also detected in the gastric ceacae and cardia (Figure 4d, e).

### DNA synthesis during *Ae. aegypti* midgut metamorphosis

**Figure 5.**
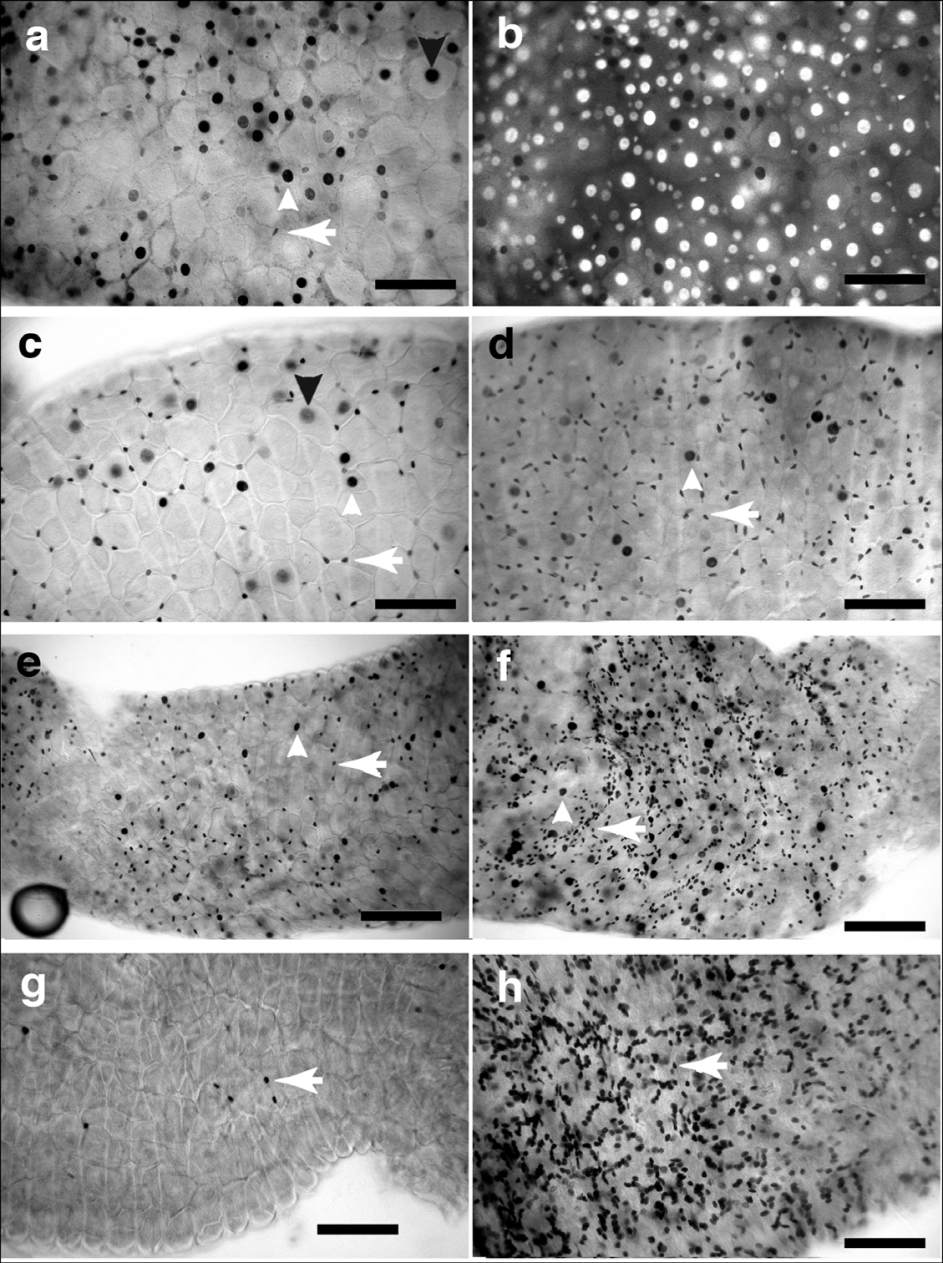
BrdU incorporation during midgut metamorphosis in Aedes *aegypti* 4^th^ instars exposed to 100 µg/ml BrdU for the time intervals indicated below. Staining was performed as described in the Methods. BrdU labeled large nuclei are indicated by a downward pointing arrow head. Intermediate sized nuclei are indicated by an upward pointing arrow head. Small oval nuclei are indicated by left pointing arrow. All images were taken at the same magnification and scale bar represents 100 µm. (a) Anterior midgut of 4^th^ instar exposed to BrdU from the time of molt to 14 hours after the molt, showing large, intermediate sized and small oval nuclei incorporated BrdU. (b) Same field as in Figure 4a but illuminated to detect DAPI staining. Most large nuclei were brightly fluorescent but not BrdU labeled, (c) Anterior midgut of 4^th^ instar exposed to BrdU from 14 to 24 hours after the molt. Large, intermediate sized and small oval nuclei incorporated BrdU. (d) Posterior midgut of 4^th^ instar exposed to BrdU from 14 to 24 hours after the molt. Incorporation by small oval nuclei greater than in Figure 5c. A few intermediate sized nuclei incorporated BrdU. (e) Anterior midgut of 4^th^ instar exposed to BrdU from 24 to 36 hours after the molt. BrdU incorporation was predominately in small oval nuclei, (f) Posterior midgut of 4^th^ instar exposed to BrdU from 24 to 36 hours after the molt. Labeled small oval nuclei were more numerous but a few intermediate sized cells were labeled, (g) Anterior midgut of 4^th^ instar exposed to BrdU from 38 to 48 hours after the molt showed very little incorporation, (h) Posterior midgut of 4^th^ instar exposed to BrdU from 38 to 48 hours after the molt showed many labeled diploid nuclei and no labeled large nuclei.

Hormonal changes occur in 4^th^ instars that signal the end of the larval phase and initiate metamorphic midgut remodeling, which includes division of diploid cells that will form the adult midgut epithelium. BrdU incorporation was used to determine when diploid cells replicated during the 4^th^ instar. During the first 24 hours after the molt to 4^th^ instars, both endoreplicating and diploid cells incorporated BrdU (Figure 5a, b, c, d). Among the endoreplicating cells, incorporation occurred mainly in those with intermediate sized nuclei. Between 24 and 36 hours after the molt the number of labeled diploid cells increased in the posterior and anterior midgut while a few endoreplicating cells incorporated BrdU (Figure 5e, f). Between 38 and 48 hours after the molt BrdU incorporation by posterior midgut diploid cells was wide spread while incorporation by anterior midgut diploid cells was sparse (Figure 5g, h). Incorporation by posterior midgut diploid cells continued until pupation, which occurred between 62 and 72 hours after the last larval-larval molt and past the time with larvae reach their maximum weight and size (data not shown).

## Discussion

### Growth of the mosquito larval midgut

The mosquito early 4^th^ instar midgut is a mixture of cells of different sizes with nuclei of different sizes (Trager 1937; Richins 1938; Richins 1945; Clements 1992). By cytological analysis, the nuclei of the larger endoreplicating cells are reported to have polytene chromosomes (Trager 1937; Sutton 1942; Gillham 1957; Clements 1992). The smallest cells are considered to have diploid nuclei because of their size as well as being associated with mitotic figures (Trager 1937). The midgut diploid cells are termed regenerative and differentiate into endoreplicating cells replacing those that were damaged and lost (Trager 1937; Richins 1938; Richins 1945). By measuring the cells of the anterior midgut in 1^st^ through 4^th^ instars, the increased size of the midgut was attributed mainly to increased cell size rather than increased cell number (Trager 1937). Our observations suggest that a substantial number of new endoreplicating cells appear during *Ae. aegypti* larval development and these postembryonic endoreplicating cells significantly contributed to the size of the midgut. Most of the growth of the *Cx. pipiens* larval anterior midgut occured by endoreplicating cell enlargement rather than by the formation of postembryonic polytene cells. However, growth of the *Cx. pipiens* posterior midgut was associated with both increased cell number and size.

Our conclusions were derived mainly from cytological observations and measurements of cells and nuclei in midgut whole mounts. A combination of hematoxylin staining and fluorescence microscopy provided a clear distinction between anterior midgut cells having the largest nuclei and cells having smaller endoreplicating or diploid nuclei that were found between the largest cells (Figure 1). Several complications had to be considered when trying to measure parameters of midgut growth during the larval phase. Whole mount microscopy of midguts provided images in which only limited regions in any one field of view were clearly focused. Measurements were restricted to the clearly focused areas and this precluded measurements from an entire anterior midgut. Although anterior and posterior midguts were unambiguously identifiable, transition areas between them were not. In 4^th^ instars the largest cells had endoreplicating nuclei that were approximately the same size as those of some smaller cells (data not shown). Thus, in *Ae. aegypti* 4^th^ instars, it was not possible to unambiguously differentiate the largest cells from some intermediate size cells by the criteria of nuclear size. The measurements were therefore limited to 1^st^, 2^nd^ and 3^rd^ instars. Measurements of 3^rd^ instar posterior midgut cells and nuclei could not be made because they were too numerous and crowded to get accurate counts. However, in *Ae. aegypti* posterior midguts, changes in number, and area, of intermediate sized cells appeared to be similar to those that occurred in anterior midguts (Figure 1).

*Ae. aegypti* 1^st^ instar anterior and posterior midgut endoreplicating cells were relatively uniform in size and distribution (Figure 1a, b). We assume that these cells uniformly grew and were the largest cells in 2^nd^ and 3^rd^ instars (Figure 1c–g). Thus an estimate of midgut growth was made by measuring the area bounded by 10 adjacent large cells. By this method it was estimated that *Ae.* *aegypti* midguts grew 5 fold between the 1^st^ and 3^rd^ instars (Figure 3a). Trager (1937) estimated that *Ae. aegypti* midguts and larvae increased in size from 1 relative unit in 1^st^ instars to 5 relative units in 3^rd^ instars.

During *Ae. aegypti* larval development there was an increase in the number of cells having small and intermediate sized nuclei that surrounded the largest cells (Figure 3c). As the area bounded by 10 adjacent large cells increased during the 1^st^ to 3^rd^ instars, the fraction of this area attributable to the small and intermediate sized cells increased from 3% in 1^st^ instars to to about 25% in 3^rd^ instars (Figure 3b). Since these cells did not appear to be present in 1^st^ instar midguts, the results suggest that a substantial fraction of larval midgut growth is attributable to an increase in cell number during the larval phase.

There was both interspecific variation, as well as midgut region variations, in the formation of intermediate sized endoreplicating cells. Using the same methods of analysis as was used with Ae. *aegypti,* it was estimated that *Cx. pipiens* anterior midguts grew approximately 4 fold between the 1^st^ to 3^rd^ instars (Figure 3a). However, the number of small and intermediate sized cells increased 2 fold during this period and the fraction of midgut area attributable to these cells was only 9% in 3^rd^ instars (Figure 3b, c). This suggests that anterior midgut growth in *Cx. pipiens* was primarily from cell enlargement. In both species the increase in intermediate sized endoreplicating cells in posterior midguts appeared to be far greater than the increase in the anterior midgut. The 4^th^ instar anterior midguts of the two species appeared very different but the posterior midguts were very similar (Figures 1h, 2h).

To explain the increased number of smaller endoreplicating cells that appeared during the larval stages we hypothesize that midgut regenerative diploid cells divide, and gave rise to mitotic diploid cells, as well as endoreplicating cells. Alternatively, 1^st^ instars may have had a population of diploid cells that were committed to the endoreplication cycle but only did so during the latter instars. It is unlikely that such cells resided in the 1^st^ instar midgut since not enough cells were observed there to account for all of the smaller endoreplicating cells in 3^rd^ or 4^th^ instar midguts. However, it is possible that new endoreplicating cells migrated into the midgut during larval development.

This hypothesis requires that midgut diploid cells regularly replicated during the early larval stages. BrdU incorporation studies showed that diploid and endoreplicating cells underwent DNA replication in 2^nd^, 3^rd^ and 4^th^ instars. The BrdU labeling pattern also suggested that all cells did not undergo DNA replication in the same time period (Figure 4). Although our results do suggest that diploid cells replicate during early instars they do not directly show that daughter diploid cells differentiate into endoreplicating cells.

### Growth of the larval midgut in other insects

The differentiation of diploid regenerative cells into midgut epithelial cells during larval development has been described in other insects. Before each molt of the apterous *Lepisma saccharina* (Zygentoma), clusters of regenerative cells give rise to columnar epithelial cells that are subsequently destroyed at the molt (Rost-Roszkowska et al. 2007). In 5^th^ instar midguts of the hemimetabolous *Locusta migratoria* there are niches of stem cells that differentiate into columnar epithelial cells and endocrine cells (Illa-Bochaca and Montuenga 2006). In the holometabolous *Manduca sexta* larval midgut stem cells divide at each molt and differentiate into epithelial cells, so that by the end of the larval phase, the dividing stem cells have given rise to a 200 fold increase in the number of larval epithelial cells (Baldwin and Hakim 1991). The arrangement of midgut imaginai histoblasts and polyploid cells of the brachycerus dipteran *Drosophila melanogaster* is very different than that of the more basal nematocerus dipteran *Ae. aegypti.* In *D. melanogaster,* midgut diploid cells begin endoreplication during embryogensis. In the 1^st^, 2^nd^ and much of the 3^rd^ instars, the midgut epithelium is composed of endoreplicating cells that are of uniform size and evenly spaced. The imaginai histoblasts are arranged in clusters, and do not replicate until just prior to pupariation. In addition, the number of endoreplicating polyploid cells does not increase during the larva phase. Metamorphic remodeling of the midgut occurs from 18 hours before pupariation to 12 hours after pupariation and involves division of midgut imaginai histoblast and autophagy of the endoreplicating cells (Madhavan and Schneiderman 1977; Zaffran et al. 1998).

### DNA synthesis during mosquito metamorphosis

Mosquito metamorphosis starts in 4^th^ instar and is completed in the pupa (Richins 1938; Richins 1945). A substantial amount of larval growth also occurs during this period (Trager 1937; Nishiura et al. 2007) so in mosquitoes there is an overlap between metamorphic changes and larval growth. Midgut metamorphosis is a remodeling process in which diploid cells divide giving rise to the adult epithelium, and the larval endoreplicating cells undergo programmed cell death. To gain a better understanding of the timing of these events and the factors that control midgut growth and remodeling, BrdU labeling was used to measure DNA replication in 4^th^ instar.

When *Ae. aegypti* 4^th^ instars were continuously fed, both endoreplicating and diploid cells synthesized DNA during the first 24 hours after the molts (Figure 5a, b, c, d). BrdU incorporation was widespread in the smaller endoreplicating cells but almost absent in the largest endoreplicating cells. Between 24 and 48 hours after the last larval-larval molt, BrdU incorporation by endoreplicating cells largely ceased while incorporation by diploid cells continued (Figure 5e, f, g, h). Posterior midgut diploid cell DNA synthesis continued in prepupae (56 hours post-molt) and the BrdU-labeled larval diploid cells were found in posterior pupal midguts (data not shown). *Ae. aegypti* larvae attain critical weight by 24 hours after the last larval-larval molt (Chambers and Klowden 1990; Nishiura et al. 2007; Telang et al. 2007). The attainment of the critical weight has been, in other insects, correlated with a drop in juvenile hormone concentration (Nijhout et al. 2006). The coincidence between the time when endoreplicating cell DNA synthesis ceased and attainment of critical weight suggests that endoreplicating cell DNA synthesis may be dependent upon the continued presence of juvenile hormone, although juvenile hormone concentrations during the mosquito larval period have not yet been determined. Diploid cell division accelerated in the posterior midgut approximately 48 hours after the molt. This time period corresponds to the time when the major ecdysone pulse occurs (Jenkins et al. 1992; Lan and Grier 2004; Telang et al. 2007).

### Growth and metamorphosis of the mosquito larval midgut

The results presented here lead to the following working hypothesis concerning *Ae. aegypti* midgut growth, development and metamorphosis. During embryonic development midgut epithelial cells begin to endocycle (Clements 1992). However, some diploid stem cells may also populate the midgut. During larval stages, we hypothesize that the diploid cells divide and some of them enter an endocycling pattern giving rise to new, postembryonic endoreplicating epithelial cells of sizes intermediate to those of the smallest diploid cells and largest endoreplicating cells. These intermediate sized cells may substantially contribute to the size and function of the larval midgut. In 4^th^ instars, we hypothesize that both the synthesis of DNA by endoreplicating cells, and the differentiation of diploid cells to endoreplicating cells, cease. Although diploid cell DNA synthesis appears to occur in 4^th^ instars of all ages, we hypothesize that the increase in ecdysone concentration that induces metamorphosis stimulates diploid cell division.

## Abbreviations

BrdU: 5-bromo-2-deoxyuridine
